# On Two Cases of Myasthenia Gravis

**Published:** 1905-12

**Authors:** J. Michell Clarke

**Affiliations:** Professor of Medicine, University College, Bristol; Physician to the Bristol General Hospital


### ON TWO CASES OF MYASTHENIA GRAVIS.

BY

J. Michell Clarke, M.A., M.D. Cantab., F.R.C.P.,

*Professor of Medicine, University College, Bristol;*
*Physician to the Bristol General Hospital.*

Before describing the two following cases of myasthenia gravis, which is also called myasthenia pseudo-paralytica, or asthenic bulbar paralysis, it may be well to recapitulate the chief features of the disease, as although much attention has been devoted to this affection, it is still somewhat unfamiliar. Very briefly stated, the chief features are weakness, amounting sometimes to complete paralysis of voluntary muscles, those in constant action being most apt to be affected, especially the eye-muscles, the cervical muscles, and those innervated from the medulla and concerned in the actions of speech, mastication, and swallowing. The muscles of the trunk and limbs are also frequently involved. The chief characteristics of the muscular affection are loss of power after voluntary contraction, and return of power after a short period of rest, except in extreme cases, and the so-called "myasthenic reaction," which is not present in every case; in this reaction the muscles affected at first respond briskly to a Faradic current, but soon cease to do so if the current continues to be passed; after a short rest, if the current is reapplied, muscular contraction again occurs and passes off, and so on. Sensation is entirely unaffected. Muscular atrophy has only been present in a very few cases. The appearance of the face is rather characteristic, with drooping upper eyelids, immobility, lack of expression, and straight line of the mouth. Gowers[1] also lays stress on an affection of smiling, which he terms the "nasal smile" or, in extreme cases, "nasal snarl." This smile consists only in elevation of the upper lip, the zygomatic muscles being inactive, with absence of the normal movement at the angles of the mouth. The speech is peculiar; it is natural at first, but on continuing it becomes nasal, and then more and more weak and indistinct, so that the words cannot be distinguished, and speech comes to a stop for want of breath. These characters are well observed by making the patient read aloud. Difficulty of swallowing gives rise to choking fits, in which several patients have died. Attacks of dyspnœa also occur without apparent cause, and are dangerous to life.

To enumerate the chief symptoms, more or fewer of which may be present in any case, these are then ptosis, diplopia, complete external ophthalmoplegia, weakness of lips, characteristic facies and speech, difficulty in mastication and swal-

[1] *Brit. M. J.*, 1902, i. 1255.

lowing, inability to hold the head upright, weakness of palate and tongue, feelings of fatigue, of aching, and stiffness of the tongue, nystagmoid jerkings of the eyes, attacks of dyspnœa, sudden giving way of the legs, a disorder of writing similar to that of speech, weakness of one or more of the limbs and of the trunk muscles. Marked remissions in the symptoms are characteristic of the course of the disease in most cases.

Hun has collected 114 published cases[1]; of these 40 were males, 72 females. The average age of onset was from 20 to 30, one only under 10 years. (In this connection the second case I report is unusually old, 63 years; Dziembowski has recorded a case in a patient of 80.) The causes, or rather the antecedents of the disease, for the cause is unknown, were in 42 cases "nervous." In 22 cases infectious diseases preceded the onset, such as tuberculosis, influenza, enteric fever, diphtheria, and in 7 toxic, as rheumatism and alcohol; in 12 there was a history of overwork. The first symptom to appear in 37 cases was ptosis, in 13 diplopia, in 16 disorders of speech, in 4 difficulty in swallowing, and in 37 paresis of the limbs. The marked tendency to variations in the course of the disease was shown in 98 cases, and in 42 there were striking remissions of months' or even years' duration.

Before discussing the pathology and diagnosis of the disease, I will now describe my cases, in which its typical features were well marked.

CASE I.—Miss W., æt. 32, no occupation, complained of debility, and that she always got very tired after doing anything.

*Family History.*—Grandfather died at 60 years of "softening of the brain"; a brother of her mother died in an asylum just over 50; her mother died of cancer of the liver; one brother died in infancy, another died of phthisis; one brother and sister are alive and well. She is the youngest of five children, her father being 49 years of age at her birth. She had influenza in 1898 and 1899, and each time was a long while in regaining strength. The present illness began after an attack of herpes zoster around the right loin.

I saw her first on October 8th, 1903, and the first symptoms began in November, 1902, when she noticed that her voice became nasal, then her tongue felt weak, and she lost control

[1] *Albany M. Ann.*, 1904, xxv. 28.

over her lips. Her chief trouble is in speech: her voice "comes through her nose," she has difficulty in speaking distinctly, and gets exhausted, so that she cannot enunciate her words. She had to give up a Sunday-school class in the spring of 1903, because her speech failed her after ten minutes' talking. About the same time she began to suffer from diplopia occasionally, chiefly for distant, not for near, vision. She has difficulty in swallowing and in masticating her food, especially in the evening, or if tired, or if she goes too long without a meal. She finds especial difficulty in eating in company, as she cannot talk during a meal. She occasionally chokes from food going the wrong way, and sometimes portions of food pass into the back of the nose. She has difficulty in swallowing saliva and in clearing her throat of mucus. She cannot whistle, and finds it difficult to blow out a candle.

Sometimes she has weakness of the hands, especially of the right hand, and cannot stretch an octave on the piano; this does not affect her writing. Sometimes she has weakness of the neck muscles, so that she cannot raise her head from the pillow without the aid of her hands. She has no pain, nor any tingling or numbness. Some time ago she tried to use a gargle, but all the fluid came out of her nose. Yesterday she had to give up reading, because the letters all ran together. All her symptoms show great variation, coming on at times and being absent at others; but they are always worse at the end of the day—for instance, she cannot smile in the evening.

On examination she is thin and poorly nourished. The facies resembles the "myopathic face": the lips are thin, the line of the mouth rather straight, the eyelids droop, giving her a sleepy, languid look. On smiling she shows the characteristic nasal smile. The face is somewhat expressionless. There is slight oscillatory lateral nystagmus. She was rather emotional, and cried a little during application of a weak Faradic current. After opening and shutting the eyelids several times they were neither so fully opened or firmly shut as at first; pupils are equal, and act well to light, accommodation, and consensually. There was diplopia, due to paresis of the left external rectus. The lips were flat and tremulous; she could not keep the lips firmly closed nor blow out the cheeks. The tongue was decidedly wasted and tremulous; she could not put it completely out, nor touch the roof of the mouth with its tip. The palatal reflex was present, but the pharynx and fauces were rather insensitive.

The muscles of the upper and lower extremities appeared normal and not wasted, the grasp of the hands feeble. All the limb reflexes were present, the knee-jerks slightly exaggerated; there was nowhere any loss of sensation.

Optic discs and retinæ normal. Fields of vision normal. No deafness. The speech was decidedly nasal; she spoke as if she had a potato in her mouth. In reading aloud her voice grew weaker and weaker and enunciation very indistinct; after reading a page breathing became hurried and laboured, and enunciation so indistinct and irregular that it was impossible to understand her. The consonants b, p, t, the soft ch, n, were the first to fail of enunciation in that order, and she lisped the consonant r.

The thoracic and abdominal viscera were normal. A few days later the electrical reactions were taken; the muscles of the lips gave a marked "myasthenic reaction" to Faradism, most on the left side. When in the evening she made an effort to smile, if she succeeded at all, it was only with the right side of the mouth. The tongue reacted fairly well. The "myasthenic reaction" was not obtained in any of the muscles of the limbs. All muscles reacted normally to the constant current, with the possible exception that for the face muscles ACC = KCC. Urine, examined on several occasions, was clear, sp. gr. 1020; no albumin or sugar; 40 oz. passed in 24 hours; urea, 1.5 to 2 per cent.; phosphoric acid ($P_2O_5$), .0495 per cent.; uric acid, .0495 per cent.; chlorides as cl., .3934 per cent.

The patient was treated in a nursing home by rest in bed, full feeding, and strychnine injections, with glycero-phosphates internally. Under these conditions she improved somewhat, but under continuous observation it was seen that her symptoms showed much variation from day to day. It was also noticed that there was not so much difference between her condition in the morning and that in the evening as when she was getting about; but, still, she had always some difficulty in eating her supper. She felt the cold very much, and when cold seemed to lose all power in her hands. After a month she took liq. thyroidea twice a day, and showed almost immediately considerable improvement; the droop of the lids improved, and after taking it a few days the diplopia disappeared for the first time since the beginning of the illness, she became able to masticate better, and had more power in biting her food; she also grew more cheerful. After six weeks' treatment she returned home, continued to rest, to take a full diet and the thyroid extract, and was somewhat better for a time.

She then went down to Weston-super-Mare, where she was under the care of Dr. Rossiter, to whom I am indebted for the following notes. Shortly after her arrival the symptoms became much worse again, especially the difficulty of swallowing, which so much increased that she was not able to take enough food to maintain her nutrition. Saliva ran away from her mouth in considerable quantity, and great discomfort was caused by the inability to swallow food and saliva; an attempt to feed her by an æsophageal catheter had to be abandoned on account of the distress it caused. She was then fed by nutrient enemata, and marked relief of symptoms and general improvement resulted from the use of hypodermic injections of morphia. This lasted eighteen days, when the enemata ceased to be retained, diarrhœa set in, and she died from exhaustion and cardiac failure. Consciousness was retained to the end, and this was not the least painful feature of the case. Death thus occurred three and a half months after I first saw her, and fourteen months after the appearance of the first slight symptoms.

CASE II.—Mrs. B., æt. 63. This patient had always enjoyed good health, and had never had any bad illness. She had always been in fairly comfortable circumstances, although she had worked hard in looking after her house. She very rarely took alcohol in any form. Her appearance was healthy for her age, she was well nourished, rather florid-looking, with some stigmata on the cheeks; the face was expressionless, and there was the right ptosis described below. Her illness began exactly one month before I saw her. She had been feeling weak and "run down" for a little time, and a month ago she noticed one day that her voice was indistinct and that she had difficulty in swallowing; it was equally difficult for her to swallow liquids or solids. Her medical man passed a stomach tube, thinking that there might be some obstruction, but the tube passed quite easily. There was no dribbling of saliva. She found that her speech varied at times, and after talking for a while it became more and more indistinct; the effort to continue speaking seemed to "hurt her in the chest," and at times to take away her breath. She had a slight cough, and began to find that she could not expectorate the phlegm. At meals mastication seemed to tire the muscles of the jaw, and she sometimes gave up eating before the end of the meal because of the fatigue of the muscles of mastication; she found especial difficulty in chewing hard substances. There were no other symptoms and no affection of the limbs, and she went on for three weeks in this state, when right ptosis came on. This was accompanied by lachrymation; during the past five days she had complained of some failure of sight, of pains in the right shoulder, and on the night before her visit to me of diplopia for some hours.

On examination she spoke indistinctly, slurring her words somewhat, and as she went on talking her speech became more indistinct; sibilants and labials were especially badly pronounced. She could not read (from lack of education), but in repeating consecutive sentences after me her articulation soon became so bad that she could hardly be understood. She had never learned to write. There was partial right ptosis, the lid being about half closed. When asked to repeatedly close the lids, the movement of the right became rapidly weaker and finally failed; the left eyelid continued to act. The oculo-motor nerves were otherwise unaffected. The pupils acted both to accommodation and light, but sluggishly. The optic discs were normal, except perhaps a trifle pale; the retinal vessels appeared healthy. Hearing normal. There was no facial paralysis. She moved the lips and tongue freely in all directions; she put out the tongue well several times in succession without fatigue. No tremor, nor wasting of the muscles of the tongue or face could be seen. The larynx was healthy, the vocal cords moved well. There was no affection of sensation over the face or mouth, unless possibly slight insensitiveness of the pharynx. The soft palate was weak, but not distinctly paralysed: it was easily fatigued. There was no affection of the limbs, neither as regards motion or sensation. The knee-jerks were normal, the elbow-jerks present, but a little difficult to obtain. Superficial reflexes normal. Nothing could be made out in the right shoulder-joint or in its neighbourhood to account for the pain there: the muscles of the right shoulder and arm showed no weakness. The thoracic and abdominal viscera and the urine presented no abnormal features. As the examination was a long one and she became rather fatigued, and was, moreover, sensitive to the Faradic current, I did not test completely for the "myasthenic reaction," but left it until a subsequent occasion. In view of the gravity of the prognosis, on account of the rapidity of onset of the symptoms, I arranged to see her again in a fortnight's time, in order to ascertain if the symptoms were still increasing, and meanwhile enjoined rest and care in feeding. I heard, however, from her medical man, Mr. C. J. Perrott, that ten days after I saw her she died quite suddenly in an access of dyspnœa. In this case the disease rapidly progressed to a fatal termination after a very short course. Unfortunately in neither case could I get leave to make a *post-mortem* examination.

With regard to the pathology of myasthenia gravis, the most striking symptoms are so evidently of bulbar origin that the failure in several fatal cases to discover any lesion in the bulb by the most modern methods of investigation excited considerable surprise. The earlier *post-mortem* reports gave negative findings, and throw no light on the origin of the disease. Gowers first suggested that the lesion might be seated in the intra-muscular nerve endings.

In 1901 Laquer and Weigert[1] published a case of the disease in which there was a tumour of the thymus gland, containing especially lymphoid cells and epithelioid formations. The muscles, normal to the naked eye, showed numerous collections of similar lymphoid cells, in the perimysium and between the muscle fibres, which were considered to be metastases. This combination of myasthenia gravis with disease of the thymus had been some time previously observed

[1] *Neurol. Centralbl.*, 1901, xx. 594.

by Weigert in a case diagnosed at that time as bulbar paralysis, but which was probably myasthenia gravis, and in one of the cases in Oppenheim's monograph on the disease there was a lymphosarcoma of the thymus.

In 1902 Goldflam[1] found in a typical case of the disease a lymphosarcoma of the right lung, with similar accumulations of lymphoid cells in the interstitial tissue of the muscle, which he also regarded as metastatic to the tumour.

Link, also in 1902,[2] reported a typical case of myasthenia gravis of five and a half months' duration, death being due to dyspnœa, in which the central nervous system was normal; but there were a persistent normal thymus and aggregations of lymphoid cells, similar to those described by Weigert and Goldflam, in several of the muscles affected by the disease during life, and macroscopically of normal appearance, *e.g.* exterior and interior recti (eyes), supinator longus, tibialis anticus and deltoids. The cells were small lymphocytes; the muscle-fibres were atrophied in the neighbourhood of the aggregations. In this case they were certainly not metastatic to a growth, nor was there any evidence of inflammation.

Similar findings have been described by Hödelmoser and Hun. In the latter's case[3] the central and peripheral nervous system was normal; the other organs were normal, except the thymus, which showed infiltration with lymphoid cells of the character of a lymphosarcoma, and the affected muscles contained collections of lymphoid cells.

Link attributed the "myasthenic symptoms" to interference with the flow of lymph in the affected muscles.

Whether the pathological changes in the above cases are to be regarded as the essential ones in myasthenia gravis it is obviously impossible to determine until further evidence is obtained. The negative findings in many fatal cases probably justify the assumption that neither in the bulb nor in the motor nerves are there any morbid changes. One would scarcely think that an enlarged or persistent thymus could be overlooked in any full *post-mortem* examination, and therefore this can

[1] *Neurol. Centralbl.*, 1902, xxi., 97, *et seq.*

[2] *Deutsche Ztschr. f. Nervenh.*, Bd. xxiii. [3] *Loc. cit*

hardly be a constant feature of the disease. The lymphoid infiltration of the muscles was in each case chronic in kind, and it has been suggested that it may have to be regarded as a secondary change due to altered circulation of the lymph in the muscles produced by the action of a toxin.

It is further interesting to note that some cases are now on record[1] in which symptoms of myasthenia gravis were associated with exophthalmic goitre. A persistent thymus has, I believe, been found in some fatal cases of exophthalmic goitre, and ophthalmoplegia externa, apart from extreme protrusion of the eyeballs, occasionally occurs in this disease.

With regard to diagnosis, it is important to remember that the first symptoms of myasthenia gravis may appear in the eye muscles, and may remain limited to them for a time before other symptoms appear. In some such cases the diagnosis has been made that the patient was suffering from that curious affection known as "migraine ophthalmique."

The strange character of many of the symptoms of myasthenia, combined sometimes with an excessive emotional tendency, has led to an erroneous diagnosis of hysteria.

In elderly or old persons with arterio-sclerosis, in whom the supply of blood to the bulbar nuclei is more or less precarious from disease of the vessels, symptoms may appear identical with those of myasthenia gravis, and often showing the same tendency to remissions, but in these cases the symptoms are confined to the bulb, do not affect the eyes, and there is a cause for them. Lastly, the occasional occurrence of bulbar symptoms, as a result of the affection of the bulbar nuclei by the diphtheritic poison, must be borne in mind.

With regard to treatment, the remarkable improvement that occurred in my case on the administration of thyroid extract, although one cannot exclude the possible coincidence of a natural remission of the disease, is worth remembering for future use. The hypodermic injection of strychnine, which has been recommended by Gowers, was also apparently of service.

[1] Meyerstein, *Neurol. Centralbl.*, 1904, xxiii. 1089; Loeser, *Ztschr. f. Augenh.*, 1904, xii. 368.

